# Circulating plasma NT-proBNP predicts subclinical coronary atherosclerosis on CT angiography among older adults in Uganda

**DOI:** 10.1186/s13104-023-06385-0

**Published:** 2023-06-19

**Authors:** Saate S Shakil, Tecla M Temu, Cissy Kityo, Geoffrey Erem MBChB MMed, Marcio S Bittencourt, Chris T Longenecker

**Affiliations:** 1 Department of Medicine, Division of Cardiology, University of California San Francisco, San Francisco, USA; 2 Department of Global Health, University of Washington Medical Center, Seattle, USA; 3 Joint Clinical Research Centre, Kampala, Uganda; 4 Department of Radiology, Makerere University, Kampala, Uganda; 5 Department of Radiology, University of Pittsburgh Medical Center, Pittsburgh, USA

**Keywords:** Cardiovascular disease, Coronary artery disease, Biomarkers, Myocardial stress

## Abstract

**Objective:**

Phenotypes and mechanisms of cardiovascular disease (CVD) may differ across global populations. In sub-Saharan Africa (SSA), distinct environmental determinants may influence development and progression of atherosclerotic coronary artery disease (CAD).

**Methods:**

We investigated associations between 6 established markers of myocardial stress and subsequent subclinical CAD (sCAD), defined as presence of any atherosclerosis on coronary CT angiography (CCTA) in a 2-year prospective cohort of Ugandan adults enriched for cardiometabolic risk factors (RFs) and HIV. Six plasma biomarkers were measured baseline among 200 participants (50% with HIV) aged ≥ 45 years with ≥ 1 cardiovascular RF. At 2-year follow-up, 132 participants (52% with HIV) who returned underwent coronary CCTA.

**Results:**

In logistic regression models adjusted for cardiovascular RFs (age, diabetes, hypertension, hyperlipidemia, smoking, obesity) and non-traditional RFs (HIV, chronic kidney disease), only NT-proBNP predicted subsequent subclinical CAD (p < 0.008, Bonferroni correction for multiple testing). In sensitivity analyses adjusted for ASCVD risk category (instead of individual RFs) in the baseline cohort with multiple imputation applied to missing year 2 CCTA data (n = 200), NT-proBNP remained significantly associated with subsequent CAD (p < 0.008).

**Conclusions:**

NT-proBNP consistently predicted subclinical CAD in Uganda in the absence of such an association among other markers of myocardial stress, suggesting a role for NT-proBNP in atherosclerosis independently of coronary microvascular dysfunction.

## Introduction

Emerging data suggest that mechanisms of cardiovascular disease (CVD), specifically coronary artery disease (CAD), may differ across global populations [1]. In sub-Saharan Africa (SSA), where CAD may be an emerging disease given the increasing burden of cardiometabolic risk factors [2, 3], distinct biological determinants of inflammation and cardiac stress may play a role in the development and progression of atherosclerosis [4, 5]. As such, there is a need for novel serum biomarkers that may be relevant for screening and surveillance of CAD in SSA, which could ultimately have global implications. We assessed relationships between serum biomarkers of cardiac stress and subsequent subclinical CAD in a 2-year prospective cohort of older persons living with HIV (PLWH) and HIV-uninfected controls in Uganda, hypothesizing that some markers of myocardial stress, specifically NT-proBNP, would be associated with subclinical coronary atherosclerosis.

## Main text

### Methods

Data used in this analysis is available from the authors upon reasonable request. We leveraged data from the Ugandan Study of HIV Effects on the Myocardium and Atherosclerosis (MUTIMA-CT) [4, 5]. This was a prospective study of 100 PLWH on antiretroviral therapy for > 6 months (with a stable regimen for > 12 weeks) and 100 age- and sex-matched HIV-uninfected controls ≥ 45 years of age in Kampala, Uganda, all with ≥ 1 major cardiovascular risk factor (hypertension, diabetes, smoking, high-density lipoprotein level < 60 mg/dL or total cholesterol ≥ 200 mg/dL [regardless of treatment status], or family history of early CAD). PLWH were recruited from the Joint Clinical Research Centre in Kampala, Uganda, while controls were recruited from local internal medicine clinics. PLWH were recruited first and then consecutive eligible HIV-negative control participants were matched by gender and age (+/- 3 years). Exclusion criteria were known history of CVD, chronic inflammatory condition, pregnancy, or use of chemotherapy/immunomodulating agents. The study was approved by the Institutional Review Boards of University Hospitals Cleveland Medical Center, the Joint Clinical Research Centre (Kampala), and by the Uganda National Council for Science and Technology. All participants provided written informed consent.

As previously described [6, 7], we measured 6 batched serum biomarkers associated with myocardial stretch, remodeling, fibrosis, and heart failure (growth differentiation factor 15 [GDF-15; ELISA, R&D Systems], galectin-3 [ELISA, R&D Systems], suppression of tumorogenicity-2 [ST-2; ELISA, R&D Systems], soluble fms-like tyrosine kinase-1 [sFLT-1; ELISA, R&D Systems], cystatin C [nephelometry, Siemens], and N-terminal pro-brain natriuretic peptide [NT-proBNP; electrochemiluminescence, Roche]) from cryopreserved plasma at -80ºC in blinded laboratories. Lower limits of detection for biomarkers were as follows: 23.4 pg/mL for GDF-15, 0.3 ng/mL for galectin-3, 31.3 pg/mL for ST-2, 31.3 pg/mL for sFLT-1, 5 pg/mL for NT-proBNP, and 0.24 mg/mL for cystatin C. Participants who returned for year 2 follow-up and had eGFR > 60 mL/min/1.73 m [2] underwent contrast coronary CT angiography (CCTA) [7]. Scans were read in batch offline by a single blinded expert reader (MSB). Subclinical CAD was assessed as a binary outcome defined as presence of detectable plaque in any evaluable coronary segments.

In primary analyses, we assessed relationships between log-transformed baseline biomarker levels (continuous) and subsequent subclinical CAD (binary) in logistic regression models adjusted for traditional cardiovascular risk factors (age [categorical; reference 45–54, 55–64, ≥ 65 years], diabetes, hypertension, total cholesterol ≥ 200 mg/dL, past or current smoking, abdominal obesity [binary; waist-to-hip ratio ≥ 0.85 for females, ≥ 0.9 for males]) and non-traditional risk enhancers (binary; HIV, chronic kidney disease [CKD] defined as urine albumin ≥ 30 mg/dL). In sensitivity analyses, we adjusted for ASCVD risk category (categorical) based on the Pooled Cohort Equations (race classified as “other”) [8], abdominal obesity, HIV, and CKD; these models were fitted to the follow-up cohort (n = 132) and the baseline cohort with multiple imputation (Mice package) applied to missing year 2 CCTA data (n = 68 lost to follow-up). Missingness was assumed to be at random and was < 5% for all predictor variables in the baseline dataset (n = 200 participants). We report odds ratios (OR), 95% confidence intervals (CI), and p-values estimated using the likelihood ratio method. Analyses were performed in RStudio 4.0.5; p < 0.008 was considered statistically significant based on a Bonferroni correction for multiple testing.

## Results and discussion

Baseline characteristics comparing PLWH and controls are presented in Table 1, while baseline and follow-up cohort characteristics are presented in Table 2. One hundred thirty-two participants who returned for year 2 CCTA were included in the primary analysis. Of these, 23 participants had detectable subclinical CAD by CCTA (prevalence 17%); 7 (30%) of these were PLWH. In multivariable models, only baseline levels of NT-proBNP were predictive of subsequent subclinical CAD in both primary and sensitivity analyses (p < 0.008 for all but ASCVD model in follow-up cohort, where p = 0.015; Table 3) in the baseline (n = 200) and follow-up (n = 132) cohort.

**Table 1:** Demographic and baseline clinical characteristics of 200 persons living with HIV (PLWH) and controls in baseline mUTIMA study cohort. Percentages/proportions are represented in parentheses. P-values are calculated using t-tests, chi-squared tests, and two-proportion z-test for continuous, categorical, and binary variables, respectively

	PLWH (n = 100)	Control (n = 100)	P (PLWH vs. Control)
Age, years (mean ± SD)	55.3 ± 6.3	56 ± 6.7	0.47
**Age group categories (n)**			0.68
45–54	50 (50)	44 (44)	
55–64	41 (41)	45 (45)	
65 +	9 (9)	11 (11)	
**ASCVD risk group (n)**			0.25
Low (< 5%)	37 (37)	25 (25)	
Borderline (5–7.5%)	20 (20)	19 (19)	
Intermediate (7.5–10%)	14 (14)	19 (19)	
High (> 10%)	29 (29)	37 (37)	
**Past Medical History (n)**			
Diabetes	26 (26)	45 (45)	0.008*
Hypertension	89 (89)	81 (81)	0.17
Current smoker	4 (4)	4 (4)	0.99
BMI			0.14
Normal	28 (28)	17 (17)	
Overweight	34 (34)	32 (32)	
Obese	36 (36)	50 (50)	
Underweight	2 (2)	1 (1)	
Waist-to-hip ratio (mean ± SD)	0.94 ± 0.12	0.89 ± 0.08	0.001*
Abdominal obesity (high WHR†)	76 (76)	63 (63)	0.07
Chronic kidney disease	15 (15)	10 (10)	0.39
GFR (mean ± SD)	98 ± 22.6	103 ± 23.6	0.14
Abnormal urine ACR ‡	30 (30)	21 (21)	0.19
High total cholesterol	58 (58)	56 (56)	0.89
High low-density lipoprotein cholesterol	51 (51)	60 (60)	0.26
Low high-density lipoprotein cholesterol	54 (54)	72 (72)	0.01*

† WHR, waist-to-hip ratio; abdominal obesity defined as WHR ≥ 0.85 for females and ≥ 0.9 for males

‡ ACR, albumin-to-creatinine ratio; abnormal: ACR ≥ 30

**Table 2:** Demographic and baseline clinical characteristics of 200 baseline and 132 follow-up study participants in the mUTIMA cohort. Percentages/proportions are represented in parentheses. P-values are calculated using t-tests, chi-squared tests, and two-proportion z-test for continuous, categorical, and binary variables, respectively

	Baseline (n = 200)	Year 2 (n = 132)	P (Baseline vs. Year 2)
Age, years (mean ± SD)	56 ± 6.5	55 ± 6.5	0.39
**Age group categories (n)**			0.80
45–54	94 (47)	67 (51)	
55–64	86 (43)	55 (42)	
65 +	20 (10)	10 (8)	
**ASCVD risk group (n)**			0.72
Low (< 5%)	62 (31)	48 (36)	
Borderline (5–7.5%)	39 (20)	26 (20)	
Intermediate (7.5–10%)	33 (17)	21 (16)	
High (> 10%)	66 (33)	37 (28)	
**Past Medical History (n)**			
Diabetes	71 (36)	40 (30)	0.39
Hypertension	170 (85)	113 (86)	0.99
Current smoker	8 (4)	7 (5)	0.77
BMI			0.93
Normal	45 (23)	31 (23)	
Overweight	66 (33)	47 (36)	
Obese	86 (43)	52 (39)	
Underweight	3 (2)	2 (2)	
Waist-to-hip ratio (mean ± SD)	0.91 ± 0.10	0.91 ± 0.09	0.71
Abdominal obesity (high WHR†)	139 (70)	93 (70)	0.95
Chronic kidney disease	25 (13)	11 (8)	0.31
GFR (mean ± SD)	100 ± 23.2	103 ± 18.8	0.19
Abnormal urine ACR ‡	51 (26)	24 (12)	0.15
HIV	100 (50)	68 (52)	0.87
High total cholesterol	114 (57)	71 (54)	0.64
High low-density lipoprotein cholesterol	111 (56)	70 (53)	0.74
Low high-density lipoprotein cholesterol	126 (63)	82 (62)	0.96

† WHR, waist-to-hip ratio; abdominal obesity defined as WHR ≥ 0.85 for females and ≥ 0.9 for males

‡ ACR, albumin-to-creatinine ratio; abnormal: ACR ≥ 30

**Table 3:** Association of each log-transformed baseline biomarker levels with presence of subsequent coronary plaque. Univariable and various multivariable models including sensitivity analyses are shown. Results are odds ratios (OR) and 95% confidence intervals (CI) per one-unit difference in log-transformed biomarker level

Log-transformed biomarker^§^	Univariable		Multivariable adjusted†		Sensitivity – ASCVD‡		Sensitivity – Imputation†*		Sensitivity – Imputation ASCVD‡*	
	OR (95% CI)	P	OR (95% CI)	P	OR (95% CI)	P	OR (95% CI)	P	OR (95% CI)	P
GDF-15	0.86 (0.47–1.47)	0.59	1.15 (0.46–2.85)	0.76	1.31 (0.55–3.11)	0.54	1.58 (0.74–3.38)	0.23	1.66 (0.81–3.38)	0.16
Galectin-3	2.67 (0.61–13.4)	0.22	3.49 (0.48–29.5)	0.23	3.33 (0.61–21.8)	0.19	4.6 (0.8–26.4)	0.09	4.86 (0.96–24.5)	0.06
ST-2	1.87 (0.58–6.26)	0.30	2.03 (0.46–8.98)	0.34	1.29 (0.35–4.88)	0.70	2.59 (0.76–8.82)	0.13	2.2 (0.75–6.46)	0.15
sFLT-1	0.92 (0.11–7.73)	0.94	0.77 (0.06–9.14)	0.84	0.75 (0.07–7.94)	0.81	2.19 (0.26–18.7)	0.47	1.38 (0.17-11)	0.76
Cystatin C	5.13 (0.62–46.7)	0.13	8.26 (0.45–182)	0.16	5.16 (0.35–91.8)	0.25	12.61 (1.34–119)	0.03	6.8 (0.75–61.6)	0.087
NT-proBNP	**1.97 (1.21–3.36)**	0.006*	**2.71 (1.39–5.95)**	0.006*	2.12 (1.2–4.06)	0.015	**2.12 (1.33–3.39)**	0.002*	**1.77 (1.19–2.63)**	0.005*

^§^ GDF-15, growth differentiation factor 15; ST-2, suppression of tumorogenicity-2; sFLT-1, soluble fms-like tyrosine kinase-1; NT-proBNP, N-terminal pro-brain natriuretic peptide

† Adjusted for age, sex, HIV, DM, HTN, high TC, past or current smoking, abdominal obesity, CKD

‡ Adjusted for ASCVD risk category, HIV, abdominal obesity, and CKD

* Models were fitted to the baseline cohort (n = 200) with multiple imputation applied to missing year 2 coronary CT angiography data

The role of NT-proBNP in atherosclerosis independently from myocardial disease remains under-investigated. Prior studies have posited that elevated plasma BNP and NT-proBNP levels may reflect subclinical coronary microvascular dysfunction in those without clinical heart failure [9]. While NT-proBNP consistently predicted subclinical CAD in our analyses, that other markers of myocardial stress did not suggests a potential role for NT-proBNP in coronary atherosclerosis that is independent of microvascular dysfunction-mediated myocardial stress. In a US-based prospective cohort of women with invasively confirmed coronary microvascular dysfunction, elevated left ventricular end diastolic pressure, and ischemic symptoms, NT-proBNP did not predict lower coronary flow reserve, suggesting a pathway for its release that is unrelated to myocardial micro-stress from distal coronary vascular dysfunction [10]. Similarly, in this Ugandan cohort, NT-proBNP’s association with subsequent subclinical CAD may be independent of microvascular dysfunction leading to myocardial stress, and could suggest a unique pathway for NT-proBNP’s involvement in coronary atherosclerosis. This hypothesis is further supported by statin trials in the antiretroviral-treated HIV population, which have shown that statin therapy decreases circulating NT-proBNP, alongside other markers involved in innate immune and novel inflammatory pathways [11]. Our findings, which align with these reports, suggest that NT-proBNP may play a role in novel inflammatory or immune mechanisms involved in the development and progression of atherosclerosis in both HIV- and non-HIV populations, which has implications for its clinical use as a screening and surveillance blood biomarker outside the heart failure population.

### Limitations

Despite the longitudinal design of our study being a strength, several key issues limit our novel findings to an exploratory rather than generalizable nature. Because our study was performed in an African population in which biological determinants of systemic biomarkers may be unique distinct from other geographic locations, our findings may not generalize to other populations, particularly those in high-income countries or those in Africa who do not have traditional risk factors for CVD. Additionally, because we leverage a cohort originally powered to investigate HIV and CVD in this setting, the substantial proportion of participants with HIV, a condition associated with immune system dysregulation, may limit extrapolation of our findings to the non-HIV population. Finally, within this small sample size, the prevalence of subclinical CAD (the outcome of interest) is low, and multicollinearity among regression variables is also possible, leading to biased effect estimates.

## Conclusions

In a prospective 2-year cohort study of Ugandan adults enriched for cardiovascular risk factors and HIV, baseline plasma NT-proBNP was predictive of subsequent subclinical coronary atherosclerosis in the absence of such an association between other markers of myocardial stress. These hypothesis-generating findings point to a possible role for NT-proBNP in atherogenesis that is independent of myocardial disease, which should be further investigated in SSA and other resource-limited regions. The exploratory findings from our small discovery study presented herein merit further investigation in larger diverse global cohorts.

## Data Availability

Due to the sensitive nature of the data used in this study and national laws governing data sharing in Uganda, the data used in this study have not been deposited in public repositories. However, data used in this study are available from the corresponding author upon reasonable request.

## Abbreviations

ASCVD: Atherosclerotic cardiovascular disease
CAD: Coronary artery disease
CCTA: Coronary computed tomography angiography
CKD: Chronic kidney disease
CVD: Cardiovascular disease
HIV: Human immunodeficiency virus
eGFR: Estimated glomerular filtration rate
NT-proBNP: N-terminal pro-brain natriuretic peptide
SSA: Sub-Saharan Africa
